# Mitochondrial DNA Profiles of Individuals from a 12th Century Necropolis in Feldioara (Transylvania)

**DOI:** 10.3390/genes12030436

**Published:** 2021-03-19

**Authors:** Alexandra Gînguță, Ioana Rusu, Cristina Mircea, Adrian Ioniță, Horia L. Banciu, Beatrice Kelemen

**Affiliations:** 1 Department of Molecular Biology and Biotechnology, Faculty of Biology and Geology, Babeș-Bolyai University, 400006 Cluj-Napoca, Romania; alexandra.ginguta@ubbcluj.ro (A.G.); cristina.mircea@ubbcluj.ro (C.M.); horia.banciu@ubbcluj.ro (H.L.B.); beatrice.kelemen@ubbcluj.ro (B.K.); 2 Molecular Biology Center, Interdisciplinary Research Institute on Bio-Nano-Sciences, Babeș-Bolyai University, 400271 Cluj-Napoca, Romania; 3 Vasile Pârvan Institute of Archaeology, Romanian Academy, 010667 Bucharest, Romania; aionita67@yahoo.com; 4 Centre for Systems Biology, Biodiversity, and Bioresources, Babeș-Bolyai University, 400006 Cluj-Napoca, Romania

**Keywords:** mitochondrial DNA, medieval individuals, Transylvania, population genetics

## Abstract

The genetic signature of modern Europeans is the cumulated result of millennia of discrete small-scale exchanges between multiple distinct population groups that performed a repeated cycle of movement, settlement, and interactions with each other. In this study we aimed to highlight one such minute genetic cycle in a sea of genetic interactions by reconstructing part of the genetic story of the migration, settlement, interaction, and legacy of what is today the Transylvanian Saxon. The analysis of the mitochondrial DNA control region of 13 medieval individuals from Feldioara necropolis (Transylvania region, Romania) reveals a genetically heterogeneous group where all identified haplotypes are different. Most of the perceived maternal lineages are of Western Eurasian origin, except for the Central Asiatic haplogroup C seen in only one sample. Comparisons with historical and modern populations describe the contribution of the investigated Saxon settlers to the genetic history of this part of Europe.

## 1. Introduction

The present-day territory of Romania reunites four distinct historical regions (Transylvania, Wallachia, Moldavia, and Dobruja), divided by the Carpathian Mountains Range that arches through the middle of the country. Until their unification into a single country in the 20th century, these provinces evolved rather independently, sometimes at odds with each other, for more than five centuries under different political and religious influences, being alternately challenged by distinct foreign powers [1]. This tangled history left its mark on the material culture, social organization, economic aspects, and genetic makeup of Romania, making it a space of convergence between different cultures. The consequences of past migrations, local admixture, and colonization events on the gene pool of current occupants of Romania’s territory have been addressed in a few studies that aimed to uncover their mitochondrial DNA (mtDNA) diversity [2,3,4]. Among these, one study assessing the overall mtDNA control region variation has indicated that the maternal genetic landscape is relatively homogenous across Romania [3]. In a different study, it has been proposed that the Romanian Carpathians do not act as a strong genetic barrier, as the mtDNA haplogroup distribution of modern populations separated by this natural boundary showed only a small degree of genetic differentiation [4]. A more detailed comparison of mtDNA variation between the historical regions pointed out that the topology of the Romanian territory might have influenced past migration routes. The present Transylvanian population stands out by being closely related to its Central European counterpart, while the populations of provinces outside the Carpathian Arch were more similar to those in the Balkans [2].

Representing, today, the central-western region of Romania, Transylvania was, in the Middle Ages, a province with a special identity within the Kingdom of Hungary, whose structure was gradually integrated during the 10th–13th centuries. This integration was carried out in stages and involved mainly two populations, known in historiography as the Szeklers and the Saxons. The first group, already existing in the kingdom since the 10th century, had a predominantly military role in defending the borders [5]. The second group arrived in Central and Eastern European territories from the Occident as part of a huge wave of colonization starting in the middle of the 12th century. Most of the Western settlers that colonized southern and southeastern Transylvania came from Flanders and Saxony, and are identified today by the generic name of Saxons [6]. This term does not designate their precise origin; it is rather used as a conventional denomination for the privileged status of the settlers from German-speaking territories. In a second stage, the Teutonic Order settled in the south-eastern corner of Transylvania [7], a region which appears under the name of Ţara Bârsei (Burzenland) and whose boundaries were established on that occasion. This land endowment, made in a complex context, had the stated purpose of protecting the southeastern borders of the kingdom against the incursions of the Cumans. In fact, it is considered that the region where these settlers arrived was less than desirable for settlement precisely because of the proximity to the Turkic nomads—the Pechenegs and Cumans. The endowment was retracted 14 years later, in 1225, as a result of the king’s dissatisfaction with the Order’s activities and especially with their tendency to act independently and to acquiesce more to the Pope than to the king. Feldioara, whose German name is Marienburg, has gone down in history mainly as the seat of the Teutonic Knights in their short Transylvanian incursion. Located approximately in the center of the territory granted to this military order, Feldioara has been founded in the middle of the 12th century by a community of German settlers (peasants and craftsmen), in a place with an important strategic position.

These colonization events imprinted the demographic history of populations from the current territory of Transylvania creating a cultural complex space, but the extent of these influences and other ancestral fine details remain largely unknown. Few studies have explored the genetic features of ethnic groups presently living in Romania in the hope of a better understanding of their biological and cultural identity, connections to other populations, and genetic legacy [8,9,10]. Most of these sought to investigate the genetic diversity of modern Hungarian-speaking groups from a maternal perspective [9], Y-chromosomal haplotypes [11], or even based on genome-wide autosomal marker data [12]. The genetic data available for modern Transylvanian Saxons is even scarcer, being limited to a single study [13]. Using Y-STR haplotype data for 59 males, it was revealed that they are genetically closer to Germans, Romanians, and Transylvanian Székely than to other European populations.

The purpose of this study is to investigate the maternal (mitochondrial) genetic diversity of medieval individuals discovered in Feldioara necropolis, potential members or descendants of a group that attained the farthest German colonization limit [14], and to consider their similarities, differences, and links to their contemporary and succeeding populations.

## 2. Materials and Methods

### 2.1. Sample Background

The human archaeological remains analyzed in this study were discovered at Feldioara necropolis, also known as Marienburg (Brașov County). The cemetery was located in a square between the evangelical church and the parsonage. The excavations in this area were conducted between 1991 and 1995 [14], in 1998, and between 2006 and 2007 [15]. A number of 127 graves with a total of 145 burials were uncovered, 109 graves outside, and 18 inside the churchyard. Of the 127 investigated graves, most were simple graves, but also multiple graves (13 double, 2 triple, and one containing the remains of four dead) were found. Of these, 14 individuals were selected for genetic analysis based on the preservation status of the osteological material (Supplementary Figure S1). The age and sex of the selected individuals were previously determined in [15]: 13 adults (6 females, 6 males, and 1 indeterminate) and 1 subadult, who had an arrowhead between the ribs on the left side of the body, presumably the cause of death [16].

The routine funerary ritual in this necropolis involved the direct deposition of the adult bodies, without coffins, in graves with a step. This type of grave consists of a first larger rectangular hole continued by a smaller inferior pit outlining the shape and size of the human body, including a niche for the head. The funeral inventory is very poor, consisting of reused Roman coins, six Hungarian anonymous denars, and three lockrings with “S” -shaped ends [15]. While this kind of lockrings are found in a larger area (from Germany [17] to the Carpathian Basin [18] in the Bjelo Brdo’s cultural space [19]) and were found in only two graves (M86, M113) in this necropolis, the anonymous Hungarian denars are minted only in the second half of the 12th century and remained in use usually for a short period of time due to frequent reminting. The six anonymous Hungarian denars found as grave goods in the Feldioara cemetery are attributed by numismatists to the reigns of Geza II (1141–1161) and Stephan III (1162–1172) and later coins were absent from the funerary inventory [14]. The beginning of use of this necropolis corresponds with the agreed start of Saxon settlement in Transylvania in the reign of Geza II and ends with the arrival of the Teutonic Knights in 1211 [14]. The stratigraphy observed during the excavation showed a layer of construction rubble covering the upper layer of the graves, originating probably in a massive edifice attributed to the Knights. Grave fills show no traces of construction debris. It is inferred that the burials at this site have been used for 2–3 generations, a fact supported by overlapping of two graves in five instances and three graves in two cases [14]. The inventory and analogies with the cemeteries of Western Europe and with those from Transylvania (all located in the Saxon colonization zone) show that the Feldioara cemetery belonged to the first wave of German settlers who arrived in Transylvania after the middle of the 12th century [14].

### 2.2. Ancient DNA Analysis

All DNA extractions and amplifications of the selected archaeological samples were performed in a sterile environment in laboratories exclusively dedicated to ancient DNA research at the Interdisciplinary Research Institute on Bio-Nano-Sciences, “Babeș-Bolyai” University, Romania. All steps were conducted following precise guidelines and appropriate workflow protocols for aDNA processing previously described [20]. Even though the persons who manipulated the samples during molecular analysis wore clean protective equipment to avoid contamination, their mtDNA signature was determined (Supplementary Table S1) and compared to the results obtained from the ancient samples.

The biological material used for DNA isolation was represented by dental samples with a very good preservation status for each tested historical individual. When available, multirooted teeth were selected. Prior to DNA extraction, each sample was decontaminated by mechanical removal of the external layer of the sample using a dental micro-drill (Marathon-3 Champion, Saeyang Microtech, Korea). Then, the samples were UV-irradiated (254 nm) for 45 min. Routinely, 100 mg of powder was collected from the dentine of the tooth root and then used for aDNA isolation. A negative control (no dental powder) was processed for every batch of three samples during the extraction and DNA amplification steps to monitor potential contamination with exogenous DNA.

The DNA extraction was performed in a designated pre-PCR area following a published silica-based protocol [21], but we adapted the described binding apparatus by using silica spin columns provided with the QIAquick PCR purification kit (Qiagen, Hilden, Germany) to allow for the recovery of products larger than 100 bp. In total, two 50 µL-TET buffer (10 mM Tris-HCL, 1 mM EDTA, 0.05% Tween-20, pH 8.0) aliquots were used for elution. The results were reinforced by at least two extractions or amplifications for several individuals, detailed in Supplementary Table S2.

The amplification of both hypervariable regions (HVR-I and HVR-II) of the human mitochondrial genome was performed using a set of eight mini-primer pairs, specially designed for highly degraded samples [22]. Besides the control region of the mitochondrial genome, three phylogenetic significant SNPs of the coding region (7028, 12308, 14766) were targeted to clarify the haplogroup classification of some samples as detailed in Supplementary Table S2, using the primers described in [23]. The amplification reactions of the targeted regions were set up in a final volume of 25 µL. Each PCR reaction consisted of 1.25 U MangoTaq™ DNA Polymerase (Bioline Reagents Ltd., London, UK), 1× MangoTaq™ Colored Reaction Buffer, 2.5 mM MgCl2, 0.2 mM dNTP mix, 0.3 µM each primer, 1–5 µL DNA extract, 0.5 µg/ µL BSA and PCR-grade water (Jena Bioscience, Jena, Germany) up to the final volume. The following cycle conditions were applied: initial denaturation at 95 °C for 5 min, 35 amplification cycles consisting of three steps (denaturation at 95 °C for 30 s, annealing at 46–56 °C depending on the primer pair, for 30 s and extension at 72 °C for 30 s) and final elongation at 72 °C for 5 min. A negative PCR control was included in each run. The PCR products were visualized on a 1.5% agarose electrophoresis gel and then purified using FavorPrep GEL and PCR Purification Kit (Favorgen Biotech Corp., Pingtung, Taiwan), according to the manufacturer’s instructions. The fragments of interest were cloned using the Sticky-End Cloning Protocol available with the CloneJet PCR Cloning Kit (Thermo Scientific, Waltham, USA) and then a batch of six clones per amplicon were Sanger-sequenced at Macrogen Europe (Amsterdam, The Netherlands) using the standard primer pJET1.2R. If these first results were inconclusive another batch of six clones per PCR fragment were generated from an independent PCR reaction. All resulting sequences were aligned using the ClustalW algorithm for multiple sequences included in BioEdit Sequence Alignment Editor v. 7.2.5.0. The mtDNA sequence polymorphisms in the nucleotide position range 57 to 357 and 16,009 to 16,390 were identified relative to the revised Cambridge Reference Sequence (rCRS, NC_012920) [24]. Haplogroup determination was carried out according to the mtDNA phylogeny of PhyloTree build 17 using the HaploGrep2 web application [25,26]. All mitochondrial control region sequences generated during this study were submitted to GenBank under the accession numbers MW508518–MW508530. The mtDNA profiles of the investigated samples are reported in Table S3.

### 2.3. Population Genetic Analyses

The mtDNA data generated for the individuals sampled in this study were compared to ancient and modern mtDNA sequences deposited in online databases to identify their genetic relatedness. This comparison aims to provide traces of evidence on past migratory routes and geographic origin of the analyzed population. The human mitochondrial sequences retrieved from publicly available data, detailed below, were first manually grouped based on their age (medieval and contemporary), and then according to their geographic origin. The resulting datasets, one consisting of 21 historical populations (including the population considered in this study) and one, including 35 modern Eurasian populations, were used for haplogroup-based analysis, as well as for sequence-based analysis as detailed below. The population from Feldioara were compared to an ancient dataset consisting of 747 sequences of European populations and a Byzantine group [27]: Lombards from Italy [28,29] and Hungary [29], Avars [30,31,32], Vikings from Norway [33] and Denmark [34], medieval Basques [35], Italians [36], Bulgarians [37], medieval population of Conquest period from Hungary [30,38], medieval populations from Poland [39], Slovakia [40], Iceland [41], southeastern Romania [20,42] and Bavaria [43]. In addition to these medieval groups, an Iron Age population attributed to Goths [44] and a population from Italy dated to the Roman period [45] were used in comparative analyses. Characterizing spatio-temporal variables and mitochondrial haplogroup frequencies are given in Table S4. For comparison with modern Eurasian diversity, we used the mtDNA haplogroup frequencies and HVR-I sequences previously compiled in Rusu et al. [20] and the references therein.

Principal component analysis (PCA) was carried out based on mtDNA haplogroup frequencies of medieval and modern datasets. In total, 33 mitochondrial haplogroups were considered in the PCA of 21 ancient populations (Supplementary Table S4), whereas in the PCA with 35 present-day Eurasian populations 37 mtDNA haplogroups were considered (detailed in [20]). All PCAs were conducted using the prcomp function of the R stats package implemented in R 4.0.2 [46], and displayed in a two-dimensional space, showing the first two principal components (PC1 and PC2). A hierarchical clustering using Ward’s agglomerative method [47] and Euclidean distance was employed on all genetic variations (PC) that resulted from the PCA of historical populations. The pvclust function in R [48] was used to calculate the probability values (*p*-values) for each cluster using 10,000 bootstrap replications. The significance of each cluster in the resulting dendrogram was given as an approximately unbiased (AU) *p*-value shown at branches.

Population comparisons using both modern and ancient reference sequences were estimated using Arlequin 3.5.2.2 [49]. Pairwise differences were calculated based on aligned HVR-I sequences of uniform sequence length, ranging from 16050 np to 16383 np (nucleotide position). The substitution model and the shape of Gamma parameter were assessed with ModelTest-NG [50]. For comparisons between the investigated population and both reference datasets, the Fst values were determined assuming a Tamura and Nei substitution model [51], 10,000 permutations and a significance level of 0.05. The linearized Slatkin Fst values and significant *p*-values of the historical populations were graphically represented as a level plot (Supplementary Table S5), while those of modern populations were visualized using multidimensional scaling (MDS) (Supplementary Table S6). The MDS plot was generated with metaMDS function based on Euclidean distances executed in the vegan library of R 4.0.2 [46,52].

## 3. Results and Discussion

### 3.1. MtDNA Polymorphism

We attempted to extract total DNA and subsequently amplify the two hypervariable regions of the human mitochondrial genome from 14 historical individuals from Feldioara. We identified the mtDNA polymorphism of the control region sequences spanning nucleotide positions 57–357 and 16009–16390 for all specimens, except one, labeled as M83a. Despite multiple extractions and amplifications for this sample with clean negative controls, the results were ambiguous, indicating potential contamination with exogenous DNA from the researcher performing the laboratory analysis. In this context, it seemed rational to exclude this sample from any further discussion and statistical analyses. The sequence motifs spanning the control region for the other 13 samples were assigned to mtDNA haplogroups in most cases with a very good overall quality of HaploGrep definitions (above 90%). The haplogroup affiliations were corroborated by diagnostic coding region sites which are all detailed in Table S3. Taking into consideration that equivalent results were obtained from replicate DNA extractions and amplifications following standard guidelines for ancient DNA analysis, as well as the detection of typical miscoding lesions in all cloned sequences support the trustworthiness of the generated mitochondrial data.

The maternal genetic composition of the analyzed group consisting of 13 samples indicates a high level of heterogeneity since it is comprised of 13 distinct haplotypes. The fact that these individuals do not share the same mtDNA profiles suggests that there are no strong maternal relationships among the analyzed group. The lack of identical maternal lineages might be a consequence of the small sample size, randomly selected from different parts of the necropolis, hardly any from adjacent burials (Supplementary Figure S1). However, other kinds of family connections cannot be ruled out solely based on the analysis of the uniparental marker that reveals the matrilineal perspective.

**Figure 1:**
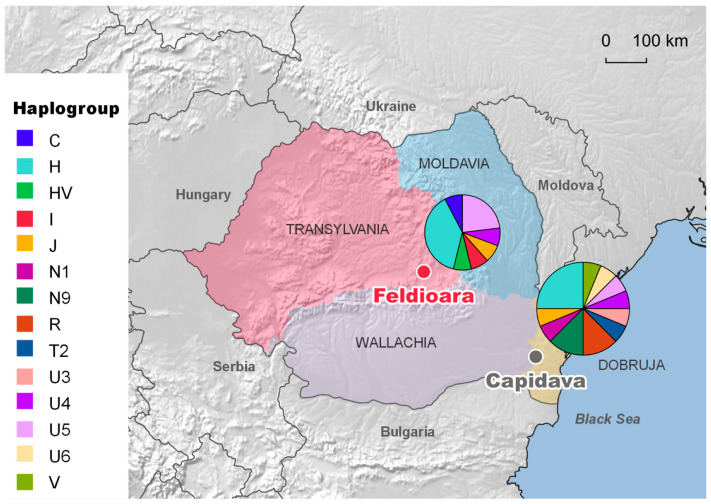
Geographic and genetic relationships between two medieval populations from the current territory of Romania: the population from Feldioara (labeled in red), located in the historical region of Transylvania, was investigated in this study, while the population from Capidava (labeled in grey, and labeled as seROU_med in further graphs and text), located in the province of Dobruja, was analyzed in previous studies [20,42]. Pie charts represent the frequencies of the major haplogroups in these populations. The size of the charts is proportional to the number of samples from each necropolis.

The mitochondrial haplogroup architecture of the analyzed medieval group includes predominantly common west Eurasian clades (H, HV, J, I, U4, and U5), but not exclusively, as one of the sequences belonging to a middle-aged female (M26) was classified into haplogroup C, an east Eurasian lineage (Figure 1). The genetic input from Central Asia has also been observed in medieval individuals from the province of Dobruja, but has been represented by a distinct mtDNA variant with a different phylogenetic history [20]. The maternal lineages originating from West Eurasia identified in the investigated population are different from those found in the small medieval group from southeastern Romania, except the common H2a2a, which may indicate different genetic influx in these geographic regions (Figure 1).

The sequence of the M26 sample showed a transition at 16357, characteristic of the C4a2 subclade. This variant is commonly found in the mtDNA pool of modern inhabitants of Siberia (particularly Evenks and Yakuts), while in Eastern Europe it is generally poorly represented [53,54]. This lineage and few derivatives were identified in ancient specimens such as: Iron Age Tagar individuals from Southern Siberia [55], Bronze Age representatives from the Baikal Lake region [56], as well as Bronze Age Kurgan specimens from the North Pontic Region [57]. C4a2a1 haplotype, distinguished by the 16171G mutation, has been identified in more recent samples dated to the medieval period, more specifically one individual from the Trans-Ural region and one attributed to Hungarian Conquerors that inhabited the Carpathian Basin during the tenth century [58]. The phylogeographic analysis of this mitochondrial lineage conducted in [58] pointed to the Kazakh or Baraba steppe as the most probable geographic origin. Based on this information and taking into account the historical context (Transylvania, which includes Feldioara, was gradually integrated into the Hungarian Kingdom during 10th–13th centuries), a feasible scenario is that this maternal lineage could have infiltrated in the Feldioara region along with the ancient Hungarians who migrated and conquered the Carpathian Basin in the 9th–10th centuries. The earliest occurrence of C4a2, until now, was documented in Early Bronze Ages samples from Shamanka (Baikal region) [59] and Middle Bronze Age samples associated with Deer Stone–Khirigsuur Complex from Mongolia [60], indicating that C4a2 most probably originated from the Mongolia–Baikal region.

Haplogroup H, the prevailing lineage in the modern gene pool of Europeans, accounts for nearly 40% of the mtDNA variation in the investigated group, being represented by different clades (H1a, H1b, H2a2a, H5, and H7f). For most of them, further classification into derivative sub-lineages might have been possible with the aid of whole coding region data, but not for H7f, which is precisely identified due to the presence of the diagnostic mutation 16168T in the reconstructed sequence of the M42a sample. This particular haplotype seems rather scarce in historical samples as it has been revealed only by the mitochondrial signature of a female from Isola Sacra associated with the Imperial Rome [61]. The M15 Feldioara sample contains defining mutations for HV9a, also identified in an individual of Viking Age from Norway, but which lacks the private mutation (16390A) present in our analyzed sample [33]. The HV9a lineage has been observed in the genomic data of Early Medieval individuals (about 500 AD.) with artificially deformed skulls discovered in present-day Bavaria, a genetically heterogeneous group comprising women that presumably originated from southeastern Europe in contrast to individuals with normal skulls from the same region that seemed to have mainly a northern and/or central European ancestry [43]. The second most abundant macro-haplogroup in the investigated population is U, with four samples classified into different subdivisions of this clade (U5b2a1a, U4a3a, and two distinct U5a1 haplotypes). Of these, U4a3a and U5a1d2a seem to be present only in few historical samples attributed to different cultures and time frames but mainly discovered in northern Europe (LM60mt—Middle Ages England [62], I2462—Early Bronze Age England [63], VK328—Viking Age Denmark [64], LP162mt—Early Medieval Denmark [62]). The genetic pattern of Feldioara sample M16a shows, besides a transition at position 207, a variant at 152 from the root node of haplogroup I, which leads to the affiliation of this individual to I2′3 lineage. A similar mtDNA profile has occurred in an ancient sample from the current territory of Germany associated with Unetice culture [65], a fact which implies a potential genetic connection between the investigated population and not only northern Europe, but also with the central part of the continent.

### 3.2. Population Genetic Analyses

**Figure 2:**
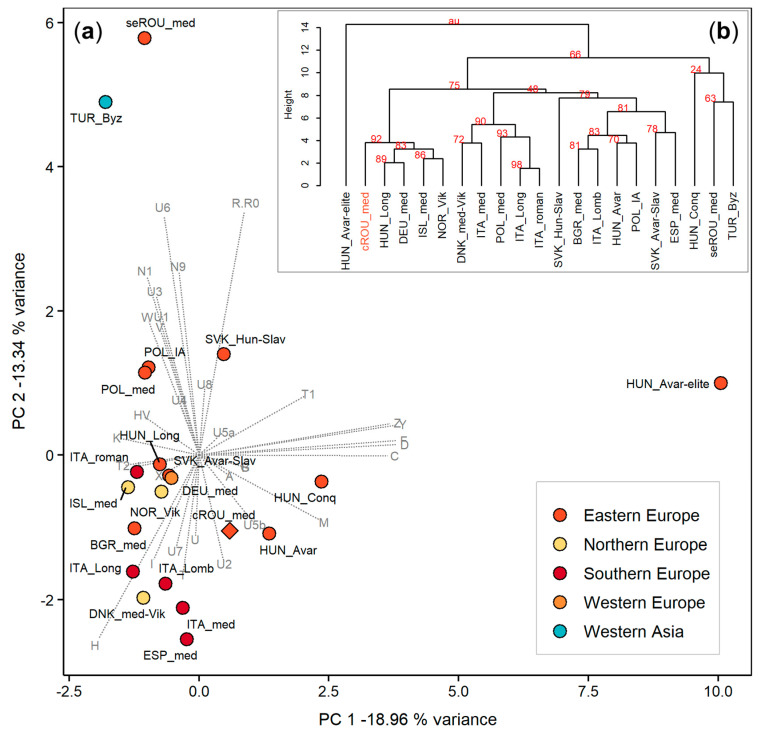
Haplogroup-based analyses of 21 historical populations. (**a**) PCA plot of the first two principal components; (**b**) Ward type hierarchical clustering (AU *p*-values in percent are given as red numbers on the dendogram). The investigated population from Feldioara (cROU_med) is clustered with early medieval individuals from Bavaria (DEU_med), Longobards from Hungary (HUN_Long), Vikings from Norway (NOR_Vik), and Early medieval Icelanders (ISL_med). This cluster is distinct from the one formed by mid-Byzantine population from southwestern Anatolia (TUR_Byz) and a medieval population from southeastern Romania (seROU_med). Abbreviations and mtDNA haplogroup frequencies are reported in Table S4.

To gain more clues regarding the genetic affinities between the sampled individuals as a group and other historical and modern populations reported in literature, we conducted haplogroup-based and sequenced-based analyses. The PCA of 21 ancient populations was constructed based on mtDNA haplogroup frequencies (Supplementary Table S4), and visually represented in a two-dimensional space (Figure 2a). The graph shows the rough clustering of most ancient European populations along the first two PCs, regardless of their geographic affiliation. Among them, only those medieval populations from southern Europe seem to be more tightly grouped. Even though the population dated to the Roman period originates from southern Italy it is separated from the South European cluster, likely due to its complex genetic background shaped by intense people interactions, imperial expansions and long-distance trades [61]. On PC1, the Avar elite group derived from the center of the Carpathian Basin (HUN_Avar-elite) is located in a marginal position. This is somehow expected as the gene pool of this group is dominated by a varied spectrum of Asian haplogroups [31]. Similarly, two isolated groups are revealed also on PC2 due to the contribution of N, R, and U suclades: a mid-Byzantine population from southwestern Anatolia (TUR_Byz) and a medieval population from southeastern Romania (seROU_med). As a consequence, the medieval population from Transylvania (cROU-med) is distantly placed from the medieval population from Dobruja, indicating distinct genetic influences in these two regions separated by the Carpathian Arch. Comparable lines of evidence were drawn by the analysis of mtDNA variation in present-day Romanians [2]. The clear separation of the two medieval populations from distinct historical provinces of Romania is also revealed by the hierarchical clustering as they are located on different branches. The Ward type hierarchical clustering (Figure 2b) also shows the genetic associations of the studied population with historical sample sets from northern Europe (Vikings from Norway and Early medieval Icelanders), early medieval burials from Bavaria, as well as Longobards from Hungary.

**Figure 3:**
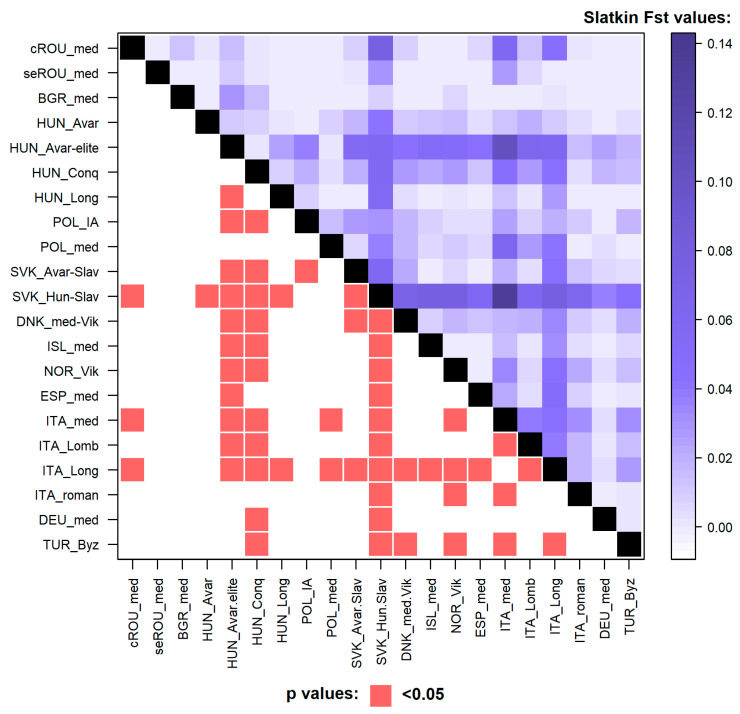
Levelplot of the linearized Slatkin population differentiation (Fst) values and significant *p*-values. Lower left corner: significant *p*-values (<0.05) are indicated in red. Upper right corner: larger Slatkin Fst values indicating greater genetic distances are marked by dark blue shades. The exact Fst and *p*-values are given in Table S5.

Pairwise genetic distances were calculated based on HVR-I sequences of uniform length using the same dataset as for the PCA. The linearized Slatkin Fst values and significant *p* values were visualized on a level plot (Figure 3). The studied population showed non-significant differences from the majority of historical populations, but not from the medieval population from a Hungarian–Slavic Contact zone, the medieval Italians, or the Longobards (also from Italy). The medieval population from Feldioara showed the lowest genetic distances from eight ancient populations of which four were placed under the same branch in the hierarchical clustering diagram (HUN_Long, DEU_med, ISL_med, and Nor_Vik). The other populations showing low genetic distances are: seROU_med, TUR_Byz, POL_med, and POL_IA. The exact Fst values and their corresponding *p*-values are listed in Table S5. Considering the small sample size analyzed in this study, the low resolution of mitochondrial control region data, and the unequal population datasets used for comparisons, these Fst results might not reflect the real genetic relationships between the compared historical populations, and as such no further interpretations and conclusions can be drawn from this data.

**Figure 4:**
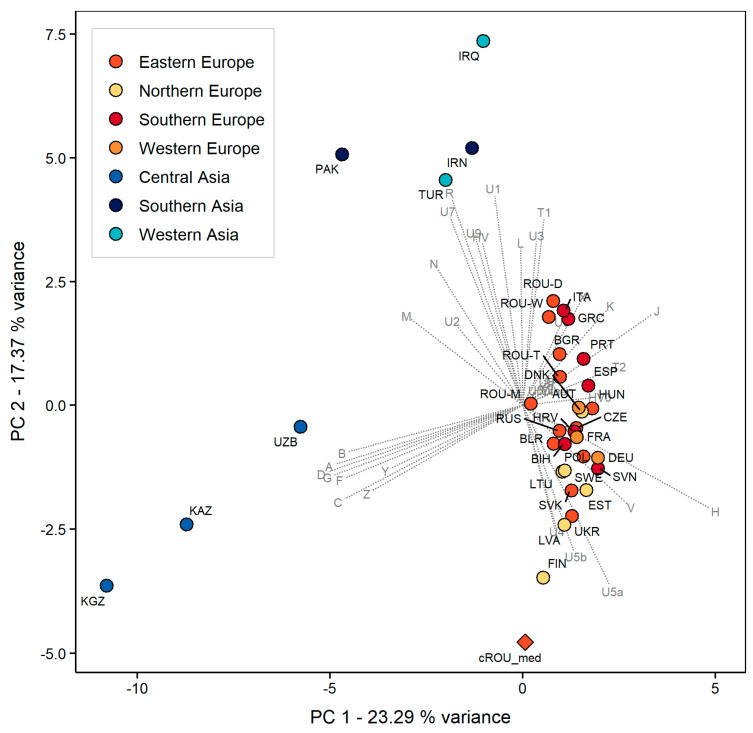
PCA plot of the investigated population (cROU_med) and modern Eurasian populations, based on mtDNA frequencies. MtDNA data for modern Eurasian populations were retrieved from previously published data in Rusu et al. 2018 [20] and references therein.

The PCA plot based on mtDNA haplogroup frequencies of modern Eurasian populations shows a fair separation of Asian and European populations along the first two components (explaining 40.66% variance) (Figure 4). The present-day European populations are more closely grouped in comparison to the Asian populations which are more dispersed. Apart from the populations originating in Northern Europe which seem to be more densely associated, no other apparent geographical grouping can be identified among European populations. The analyzed population from Transylvania is located on the verge of the European populations’ cluster on PC2 and has a higher genetic affinity to some Slavic populations (Ukraine and Slovakia) and modern populations of the Baltic region (Finland, Latvia, and Estonia) than to the others. Most probably, the association of the Feldioara population to the Northern European ones, particularly to modern Finns, is promoted by the relatively high frequency of U lineages in the maternal genetic composition of the investigated group, also present in notable proportions in northern Fenno-Scandinavia and the East Baltic region [66].

**Figure 5:**
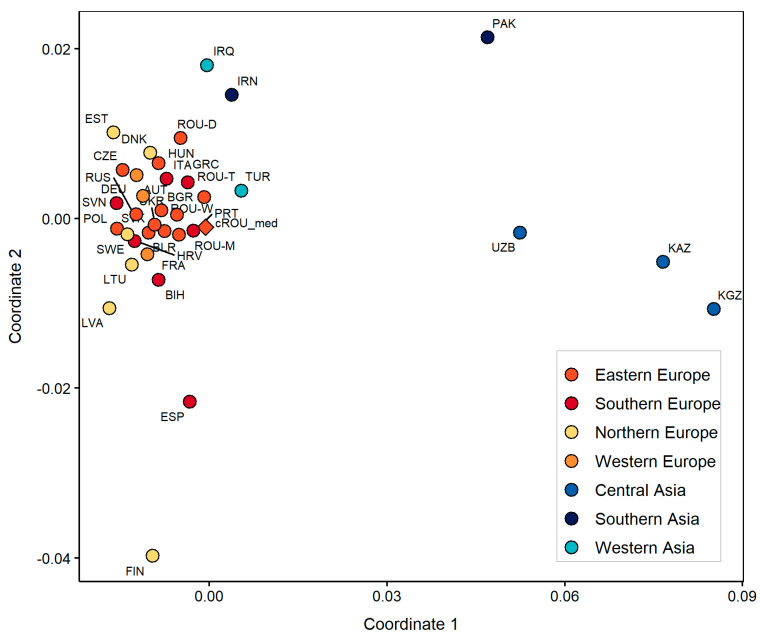
MDS plot of the investigated population (cROU_med) and modern Eurasian populations. The MDS plot was performed using linearized Slatkin Fst values, detailed in Table S6. Stress value is 0.1085145.

The medieval Feldioara population showed significant differences (*p* < 0.05) from two modern populations located in Central Asia (Kazakhstan and Kyrgyzstan), while the lowest genetic distances were observed relative to contemporary Slavic populations, mainly from Eastern Europe (BLR, SVK, UKR, LTU, and RUS). The calculated Fst values between pairs of populations and their corresponding *p*-values, along with reference population information are given in Table S6. The output of the genetic distance analysis was plotted by a MDS (Figure 5). The distribution pattern of modern Eurasian populations reflected by the MDS plot resembles the image of the PCA as most of the European populations are closely grouped, whereas the Asian ones are more scattered along the first two coordinates. The cROU_med is placed in the proximity of modern Romanians from Transylvania and of its two adjacent historical regions of Romania (Wallachia and Moldavia). In contrast, the present-day population from Dobruja, the most geographically distant territory from Transylvania, is less genetically connected to the population investigated in this study. However, the genetic distances between the investigated population and modern Eurasian populations do not allow for unequivocal conclusions as the resulting configuration is sketched only by a partial marker in the genome and is influenced by the low sample size of Feldioara group.

## 4. Conclusions

To gain insights into the genetic relatedness of a Middle Age population of central Romania, we have successfully investigated the mtDNA of 13 individuals excavated from the 12th–13th century necropolis of Feldioara. All 13 individuals analyzed in this study display a different mitochondrial lineage, reflecting a lack of close maternal relationships, even between individuals interred closely by one another. This kind of diversity may characterize a relatively recently established group, with high input of individuals with different geographic origins. West Eurasian haplogroups (H, HV, J, I, U4, and U5) dominate among the sampled individuals, the exception being haplogroup C. Based on available published data, the C4a2 variant identified in one Feldioara sample, can be ultimately traced to the Eastern Asian Bronze Age. A possible scenario of the influx of this lineage into Feldioara might be related to the arrival of Hungarian conquerors in the Carpathian Basin at the end of the 9th century. The population genetic comparisons performed in this study potentially reveal some connections to other historical or modern Eurasian population, but these relations should be treated with care due to the low resolution of the emerging pattern shaped by the low samples size and the use of a partial mtDNA genomic marker. A much more complex and accurate picture of ancestry for the Transylvanian region might be provided by future genome-wide studies and increased number of samples.

## Data Availability

The mtDNA sequences presented in this study were submitted to GenBank (https://www.ncbi.nlm.nih.gov/genbank/) under the accession numbers MW508518-MW508530. All other relevant data are within the paper and its Supplementary Materials.

## Supplementary Materials

The following are available online at https://www.mdpi.com/2073-4425/12/3/436/s1, Figure S1: Overview of graves distribution within Feldioara necropolis (simplified from [14]), Table S1: MtDNA profile of the researchers, Table S2: Samples—processing details, Table S3: MtDNA profile of the investigated samples, Table S4: MtDNA haplogroup frequencies used for PCA with 21 historical populations, Table S5: Fst values, *p*-values and Slatkin Fst matrix of the 21 historical populations, Table S6: Fst values, *p*-values and Slatkin Fst matrix of the medieval population from Feldioara and modern Eurasian populations.

## References

[1] Curta F. (2006). Southeastern Europe in the Middle Ages, 500–1250.

[2] Cocos R., Schipor S., Hervella M., Cianga P., Popescu R., Banescu C., Constantinescu M., Martinescu A., Raicu F. (2017). Genetic affinities among the historical provinces of Romania and Central Europe as revealed by an mtDNA analysis. BMC Genet..

[3] Turchi C., Stanciu F., Paselli G., Buscemi L., Parson W., Tagliabracci A. (2016). The mitochondrial DNA makeup of Romanians: A forensic mtDNA control region database and phylogenetic characterization. Forensic Sci. Int. Genet..

[4] Hervella M., Izagirre N., Alonso S., Ioana M., Netea M.G., de-la-Rua C. (2014). The Carpathian range represents a weak genetic barrier in South-East Europe. BMC Genet..

[5] Göckenjan H. (1972). Hilfsvölker und Grenzwächter im Mittelalterlichen Ungarn.

[6] Nägler T. (1979). Die Ansiedlung der Siebenbürger Sachsen.

[7] Zimmermann H. (2011). Der Deutsche Orden in Siebenbürgen: Eine Diplomatische Untersuchung.

[8] Bosch E., Calafell F., Gonzalez-Neira A., Flaiz C., Mateu E., Scheil H.G., Huckenbeck W., Efremovska L., Mikerezi I., Xirotiris N. (2006). Paternal and maternal lineages in the Balkans show a homogeneous landscape over linguistic barriers, except for the isolated Aromuns. Ann. Hum. Genet..

[9] Brandstatter A., Egyed B., Zimmermann B., Duftner N., Padar Z., Parson W. (2007). Migration rates and genetic structure of two Hungarian ethnic groups in Transylvania, Romania. Ann. Hum. Genet..

[10] Mendizabal I., Lao O., Marigorta U.M., Wollstein A., Gusmao L., Ferak V., Ioana M., Jordanova A., Kaneva R., Kouvatsi A. (2012). Reconstructing the population history of European Romani from genome-wide data. Curr. Biol. CB.

[11] Csányi B., Bogácsi-Szabó E., Tömöry G., Czibula Á., Priskin K., Csõsz A., Mende B., Langó P., Csete K., Zsolnai A. (2008). Y-Chromosome Analysis of Ancient Hungarian and Two Modern Hungarian-Speaking Populations from the Carpathian Basin. Ann. Hum. Genet..

[12] Ádám V., Bánfai Z., Maász A., Sümegi K., Miseta A., Melegh B. (2020). Investigating the genetic characteristics of the Csangos, a traditionally Hungarian speaking ethnic group residing in Romania. J. Hum. Genet..

[13] Barbarii L., Constantinescu C., Rolf B. (2004). A study on Y-STR haplotypes in the Saxon population from Transylvania (Siebenburger Sachsen): Is there an evidence for a German origin?. Rom. J. Leg. Med..

[14] Ioniță A., Căpățână D., Boroffka N.G.O., Boroffka R., Popescu A. (2004). Feldioara-Marienburg: Contribuții Arheologice la istoria Țării Bârsei: Archaölogische Beiträge zur Geschichte des Burzenlandes.

[15] Muja C., Ioniță A. (2015). Sexual dimorphism and general activity levels as revealed by the diaphyseal external shape and historical evidence: Case study on a medieval population from Transylvania. Dacia N.S..

[16] Ioniță A. (2013). Die Besiedlung des Burzenlandes im 12.-13. Jahrhundert im Lichte der Archäologie. Generalprobe Burzenland.

[17] REMPEL H. (1966). Reihengräberfriedhöfe des 8. bis 11. Jahrhunderts aus Sachsen-Anhalt, Sachsen und Thüringen. Deutsche Akademie der Wissenschaften zu Berlin. Schr. Der Sekt. Für Vor-U. Frühgeschichte.

[18] Gáll E. (2009). The date of the appearance of the S-ended lock-rings in the Transylvanian Basin. Ephemer. Napoc..

[19] Giesler J. (1981). Untersuchungen zur chronologie der Bijelo Brdo-Kultur. Ein beitrag zur archäologie des 10. und 11. jahrhunderts im Karpatenbecken. Prähistorische Z..

[20] Rusu I., Modi A., Vai S., Pilli E., Mircea C., Radu C., Urduzia C., Pinter Z.K., Bodolica V., Dobrinescu C. (2018). Maternal DNA lineages at the gate of Europe in the 10^th^ century AD. PLoS ONE.

[21] Dabney J., Knapp M., Glocke I., Gansauge M.T., Weihmann A., Nickel B., Valdiosera C., Garcia N., Paabo S., Arsuaga J.L. (2013). Complete mitochondrial genome sequence of a Middle Pleistocene cave bear reconstructed from ultrashort DNA fragments. Proc. Natl. Acad. Sci. USA.

[22] Gabriel M.N., Huffine E.F., Ryan J.H., Holland M.M., Parsons T.J. (2001). Improved MtDNA sequence analysis of forensic remains using a "mini-primer set" amplification strategy. J. Forensic Sci..

[23] Tömöry G., Csányi B., Bogácsi-Szabó E., Kalmár T., Czibula A., Csősz A., Priskin K., Mende B., Langó P., Downes C.S. (2007). Comparison of maternal lineage and biogeographic analyses of ancient and modern Hungarian populations. Am. J. Phys. Anthropol..

[24] Andrews R.M., Kubacka I., Chinnery P.F., Lightowlers R.N., Turnbull D.M., Howell N. (1999). Reanalysis and revision of the Cambridge reference sequence for human mitochondrial DNA. Nat. Genet..

[25] van Oven M. (2015). PhyloTree Build 17: Growing the human mitochondrial DNA tree. Forensic Sci. Int. Genet. Suppl. Ser..

[26] Weissensteiner H., Pacher D., Kloss-Brandstatter A., Forer L., Specht G., Bandelt H.J., Kronenberg F., Salas A., Schonherr S. (2016). HaploGrep 2: Mitochondrial haplogroup classification in the era of high-throughput sequencing. Nucleic Acids Res..

[27] Ottoni C., Ricaut F.X., Vanderheyden N., Brucato N., Waelkens M., Decorte R. (2011). Mitochondrial analysis of a Byzantine population reveals the differential impact of multiple historical events in South Anatolia. Eur. J. Hum. Genet. Ejhg.

[28] Vai S., Ghirotto S., Pilli E., Tassi F., Lari M., Rizzi E., Matas-Lalueza L., Ramirez O., Lalueza-Fox C., Achilli A. (2015). Genealogical relationships between early medieval and modern inhabitants of Piedmont. PLoS ONE.

[29] Vai S., Brunelli A., Modi A., Tassi F., Vergata C., Pilli E., Lari M., Susca R.R., Giostra C., Baricco L.P. (2019). A genetic perspective on Longobard-Era migrations. Eur. J. Hum. Genet..

[30] Csősz A., Szécsényi-Nagy A., Csákyová V., Langó P., Bódis V., Köhler K., Tömöry G., Nagy M., Mende B.G. (2016). Maternal Genetic Ancestry and Legacy of 10(th) Century AD Hungarians. Sci. Rep..

[31] Csáky V., Gerber D., Koncz I., Csiky G., Mende B.G., Szeifert B., Egyed B., Pamjav H., Marcsik A., Molnár E. (2020). Genetic insights into the social organisation of the Avar period elite in the 7th century AD Carpathian Basin. Sci. Rep..

[32] Šebest L., Baldovič M., Frtús A., Bognár C., Kyselicová K., Kádasi Ľ., Beňuš R. (2018). Detection of mitochondrial haplogroups in a small avar-slavic population from the eigth–ninth century AD. Am. J. Phys. Anthropol..

[33] Krzewińska M., Bjornstad G., Skoglund P., Olason P.I., Bill J., Götherström A., Hagelberg E. (2015). Mitochondrial DNA variation in the Viking age population of Norway. Philos. Trans. R. Soc. Lond. Ser. B Biol. Sci..

[34] Melchior L., Lynnerup N., Siegismund H.R., Kivisild T., Dissing J. (2010). Genetic diversity among ancient Nordic populations. PLoS ONE.

[35] Alzualde A., Izagirre N., Alonso S., Alonso A., Albarrán C., Azkarate A., de la Rúa C. (2006). Insights into the "isolation" of the Basques: mtDNA lineages from the historical site of Aldaieta (6th-7th centuries AD). Am. J. Phys. Anthropol..

[36] Guimaraes S., Ghirotto S., Benazzo A., Milani L., Lari M., Pilli E., Pecchioli E., Mallegni F., Lippi B., Bertoldi F. (2009). Genealogical discontinuities among Etruscan, Medieval, and contemporary Tuscans. Mol. Biol. Evol..

[37] Nesheva D.V., Karachanak-Yankova S., Lari M., Yordanov Y., Galabov A., Caramelli D., Toncheva D. (2015). Mitochondrial DNA Suggests a Western Eurasian Origin for Ancient (Proto-) Bulgarians. Hum. Biol..

[38] Neparaczki E., Maroti Z., Kalmar T., Kocsy K., Maar K., Bihari P., Nagy I., Fothi E., Pap I., Kustar A. (2018). Mitogenomic data indicate admixture components of Central-Inner Asian and Srubnaya origin in the conquering Hungarians. PLoS ONE.

[39] Juras A., Dabert M., Kushniarevich A., Malmström H., Raghavan M., Kosicki J.Z., Metspalu E., Willerslev E., Piontek J. (2014). Ancient DNA reveals matrilineal continuity in present-day Poland over the last two millennia. PLoS ONE.

[40] Csákyová V., Szécsényi-Nagy A., Csősz A., Nagy M., Fusek G., Langó P., Bauer M., Mende B.G., Makovický P., Bauerová M. (2016). Maternal Genetic Composition of a Medieval Population from a Hungarian-Slavic Contact Zone in Central Europe. PLoS ONE.

[41] Helgason A., Lalueza-Fox C., Ghosh S., Sigurethardóttir S., Sampietro M.L., Gigli E., Baker A., Bertranpetit J., Arnadóttir L., Thornorsteinsdottir U. (2009). Sequences from first settlers reveal rapid evolution in Icelandic mtDNA pool. Plos Genet..

[42] Rusu I., Modi A., Radu C., Mircea C., Vulpoi A., Dobrinescu C., Bodolică V., Potârniche T., Popescu O., Caramelli D. (2019). Mitochondrial ancestry of medieval individuals carelessly interred in a multiple burial from southeastern Romania. Sci. Rep..

[43] Veeramah K.R., Rott A., Groß M., van Dorp L., López S., Kirsanow K., Sell C., Blöcher J., Wegmann D., Link V. (2018). Population genomic analysis of elongated skulls reveals extensive female-biased immigration in Early Medieval Bavaria. Proc. Natl. Acad. Sci. USA.

[44] Stolarek I., Handschuh L., Juras A., Nowaczewska W., Kóčka-Krenz H., Michalowski A., Piontek J., Kozlowski P., Figlerowicz M. (2019). Goth migration induced changes in the matrilineal genetic structure of the central-east European population. Sci. Rep..

[45] Emery M.V., Duggan A.T., Murchie T.J., Stark R.J., Klunk J., Hider J., Eaton K., Karpinski E., Schwarcz H.P., Poinar H.N. (2018). Ancient Roman mitochondrial genomes and isotopes reveal relationships and geographic origins at the local and pan-Mediterranean scales. J. Archaeol. Sci. Rep..

[46] R Core T. (2020). R: A Language and Environment for Statistical Computing.

[47] Ward J.H. (1963). Hierarchical grouping to optimize an objective function. J. Am. Stat. Assoc..

[48] Suzuki R., Terada Y., Shimodaira H. (2019). pvclust: Hierarchical Clustering with P-Values via Multiscale Bootstrap Resampling.

[49] Excoffier L., Lischer H.E. (2010). Arlequin suite ver 3.5: A new series of programs to perform population genetics analyses under Linux and Windows. Mol. Ecol. Resour..

[50] Darriba D., Posada D., Kozlov A.M., Stamatakis A., Morel B., Flouri T. (2019). ModelTest-NG: A New and Scalable Tool for the Selection of DNA and Protein Evolutionary Models. Mol. Biol. Evol..

[51] Tamura K., Nei M. (1993). Estimation of the number of nucleotide substitutions in the control region of mitochondrial DNA in humans and chimpanzees. Mol. Biol. Evol..

[52] Oksanen J., Blanchet F.G., Friendly M., Kindt R., Legendre P., McGlinn D., Minchin P.R., O’Hara R.B., Simpson G.L., Solymos P. (2019). Vegan: Community Ecology Package.

[53] Nikitin A.G., Newton J.R., Potekhina I.D. (2012). Mitochondrial haplogroup C in ancient mitochondrial DNA from Ukraine extends the presence of East Eurasian genetic lineages in Neolithic Central and Eastern Europe. J. Hum. Genet..

[54] Duggan A.T., Whitten M., Wiebe V., Crawford M., Butthof A., Spitsyn V., Makarov S., Novgorodov I., Osakovsky V., Pakendorf B. (2013). Investigating the Prehistory of Tungusic Peoples of Siberia and the Amur-Ussuri Region with Complete mtDNA Genome Sequences and Y-chromosomal Markers. PLoS ONE.

[55] Pilipenko A.S., Trapezov R.O., Cherdantsev S.V., Babenko V.N., Nesterova M.S., Pozdnyakov D.V., Molodin V.I., Polosmak N.V. (2018). Maternal genetic features of the Iron Age Tagar population from Southern Siberia (1st millennium BC). PLoS ONE.

[56] de Barros Damgaard P., Martiniano R., Kamm J., Moreno-Mayar J.V., Kroonen G., Peyrot M., Barjamovic G., Rasmussen S., Zacho C., Baimukhanov N. (2018). The first horse herders and the impact of early Bronze Age steppe expansions into Asia. Science.

[57] Nikitin A.G., Ivanova S., Kiosak D., Badgerow J., Pashnick J. (2017). Subdivisions of haplogroups U and C encompass mitochondrial DNA lineages of Eneolithic–Early Bronze Age Kurgan populations of western North Pontic steppe. J. Hum. Genet..

[58] Csáky V., Gerber D., Szeifert B., Egyed B., Stégmár B., Botalov S.G.e., Grudochko I.V.e., Matveeva N.P., Zelenkov A.S., Sleptsova A.V. (2020). Early medieval genetic data from Ural region evaluated in the light of archaeological evidence of ancient Hungarians. Sci. Rep..

[59] Damgaard P.d.B., Marchi N., Rasmussen S., Peyrot M., Renaud G., Korneliussen T., Moreno-Mayar J.V., Pedersen M.W., Goldberg A., Usmanova E. (2018). 137 ancient human genomes from across the Eurasian steppes. Nature.

[60] Jeong C., Wang K., Wilkin S., Taylor W.T.T., Miller B.K., Bemmann J.H., Stahl R., Chiovelli C., Knolle F., Ulziibayar S. (2020). A Dynamic 6,000-Year Genetic History of Eurasia’s Eastern Steppe. Cell.

[61] Antonio M.L., Gao Z., Moots H.M., Lucci M., Candilio F., Sawyer S., Oberreiter V., Calderon D., Devitofranceschi K., Aikens R.C. (2019). Ancient Rome: A genetic crossroads of Europe and the Mediterranean. Science.

[62] Klunk J., Duggan A.T., Redfern R., Gamble J., Boldsen J.L., Golding G.B., Walter B.S., Eaton K., Stangroom J., Rouillard J.-M. (2019). Genetic resiliency and the Black Death: No apparent loss of mitogenomic diversity due to the Black Death in medieval London and Denmark. Am. J. Phys. Anthropol..

[63] Olalde I., Brace S., Allentoft M.E., Armit I., Kristiansen K., Booth T., Rohland N., Mallick S., Szecsenyi-Nagy A., Mittnik A. (2018). The Beaker phenomenon and the genomic transformation of northwest Europe. Nature.

[64] Margaryan A., Lawson D.J., Sikora M., Racimo F., Rasmussen S., Moltke I., Cassidy L.M., Jørsboe E., Ingason A., Pedersen M.W. (2020). Population genomics of the Viking world. Nature.

[65] Mathieson I., Lazaridis I., Rohland N., Mallick S., Patterson N., Roodenberg S.A., Harney E., Stewardson K., Fernandes D., Novak M. (2015). Genome-wide patterns of selection in 230 ancient Eurasians. Nature.

[66] Malyarchuk B., Derenko M., Grzybowski T., Perkova M., Rogalla U., Vanecek T., Tsybovsky I. (2010). The peopling of Europe from the mitochondrial haplogroup U5 perspective. PLoS ONE.

